# The 5-formyltetrahydrofolate futile cycle reduces pathway stochasticity in an extended hybrid-stochastic model of folate-mediated one-carbon metabolism

**DOI:** 10.1038/s41598-019-40230-4

**Published:** 2019-03-13

**Authors:** Karla Misselbeck, Luca Marchetti, Corrado Priami, Patrick J. Stover, Martha S. Field

**Affiliations:** 1 The Microsoft Research - University of Trento Centre for Computational and Systems Biology (COSBI), Piazza Manifattura, 1, 38068 Rovereto, Italy; 2 Department of Mathematics, University of Trento, 38123 Povo, Italy; 3 Department of Computer Science, University of Pisa, 56127 Pisa, Italy; 4 College of Agriculture and Life Sciences, Texas A&M University, College Station, TX 77843-2142 USA; 5 Division of Nutritional Sciences, Cornell University, Ithaca, NY 14853 USA

## Abstract

In folate-mediated one-carbon metabolism (FOCM), 5-formyltetrahydrofolate (5fTHF), a one-carbon substituted tetrahydrofolate (THF) vitamer, acts as an intracellular storage form of folate and as an inhibitor of the folate-dependent enzymes phosphoribosylaminoimidazolecarboxamide formyltransferase (AICARFT) and serine hydroxymethyltransferase (SHMT). Cellular levels of 5fTHF are regulated by a futile cycle comprising the enzymes SHMT and 5,10-methenyltetrahydrofolate synthetase (MTHFS). MTHFS is an essential gene in mice; however, the roles of both 5fTHF and MTHFS in mammalian FOCM remain to be fully elucidated. We present an extension of our previously published hybrid-stochastic model of FOCM by including the 5fTHF futile-cycle to explore its effect on the FOCM network. Model simulations indicate that MTHFS plays an essential role in preventing 5fTHF accumulation, which consequently averts inhibition of all other reactions in the metabolic network. Moreover, *in silico* experiments show that 10-formylTHF inhibition of MTHFS is critical for regulating purine synthesis. Model simulations also provide evidence that 5-methylTHF (and not 5fTHF) is the predominant physiological binder/inhibitor of SHMT. Finally, the model simulations indicate that the 5fTHF futile cycle dampens the stochastic noise in FOCM that results from both folate deficiency and a common variant in the methylenetetrahydrofolate reductase (*MTHFR*) gene.

## Introduction

Folate-mediated one-carbon metabolism (FOCM, for a list of the abbreviations refer to Supplementary Table 1) is a tightly interconnected metabolic network in which tetrahydrofolates (THF) carry and chemically activate one-carbon moieties for biosynthetic reactions including *de novo* purine synthesis, *de novo* thymidylate (dTMP) synthesis, and remethylation of homocysteine to methionine^1^. Methionine is a precursor of S-adenosylmethionine (SAM), which serves as a methyl donor for DNA methylation, protein methylation, and lipid and neurotransmitter synthesis. Biomarkers of impaired FOCM include uracil misincorporation into DNA, DNA damage, and increased plasma homocysteine^2^. Impaired FOCM is also associated with increased risk for neural tube defects^3^, development of certain types of cancer^4–6^, and neurodegenerative diseases^7^. However, causal relationships between biomarkers of impaired FOCM and development of pathology remain unresolved.

There are five one-carbon substituted THF derivatives *in vivo*, and these derivatives carry one-carbon units at one of three oxidation levels ranging from formate to methanol, with each substituted folate serving in unique one-carbon transfer reactions. 10-formylTHF (10fTHF), 5-formylTHF (5fTHF), and 5,10-methenylTHF (CHF) carry one-carbon units at the oxidation state of formate. The formyl group of 10fTHF, the folate co-factor used by phosphoribosylaminoimidazolecarboxamide formyltransferase (AICARFT) and phosphoribosylglycinamide formyltransferase (PGT), is incorporated into the #2 and #8 carbons of the purine ring. 10fTHF is formed from THF and ATP by the synthetase activity of the trifunctional enzyme methylenetetrahydrofolate dehydrogenase 1 (MTHFD1). 5fTHF is not used as a cofactor for folate-dependent biosynthetic reactions, rather it is thought to be an intracellular storage form of folate in dormant cells^8^. 5fTHF is regulated through a futile cycle catalyzed by serine hydroxymethyltransferase (SHMT) and methenyltetrahydrofolate synthetase (MTHFS). 5fTHF is generated from CHF in an irreversible reaction catalyzed by SHMT. 5fTHF is re-introduced to the folate cofactor pool by MTHFS, which converts 5fTHF to CHF in an irreversible, ATP-dependent reaction. CHF is not used directly as a co-factor for biosynthetic reactions. It is formed both enzymatically from MTHFS and the cyclohydrolase activity of MTHFD1 and non-enzymatically from both 5fTHF and 10fTHF^9^.

MTHFS is an essential gene in mice^10^, but its role in mammalian FOCM remains to be fully elucidated. Increased MTHFS expression in cultured cells shifts the distribution of folate cofactors toward 10fTHF at the expense of 5methylTHF (5mTHF) and also leads to increased rates of folate catabolism^11^, presumably by shifting the folate distribution in favor of the accumulation of more chemically unstable forms of folate. 10fTHF binds tightly to and inhibits MTHFS *in vitro*^12,13^, and increased MTHFS expression in cultured cells leads to increased rates of *de novo* purine biosynthesis^13^. MTHFS expression in cultured cells also decreases efficacy of anti-folate chemotherapeutic agents designed to target *de novo* purine synthesis^14^. MTHFS physically interacts with the “purinosome,” a multi-enzyme complex that forms under purine-deficient conditions and consists of the six enzymes required for *de novo* purine synthesis. It has been suggested that MTHFS serves to channel 10fTHF cofactors to the purinosome^10^.

The SHMT- and MTHFS-catalyzed “futile cycle” may serve regulatory functions by controlling 5fTHF concentrations. The primary metabolic function of SHMT is to reversibly interconvert serine and THF to glycine and 5,10-methyleneTHF (CH2F). 5fTHF is a feedback inhibitor of SHMT, and also binds to and inhibits AICARFT^15,16^, but the purpose of the 5fTHF futile cycle in regulating SHMT and FOCM remains unresolved. This is due in part because 5mTHF, which is more abundant than 5fTHF, also serves as a potent inhibitor of SHMT.

**Figure 1:**
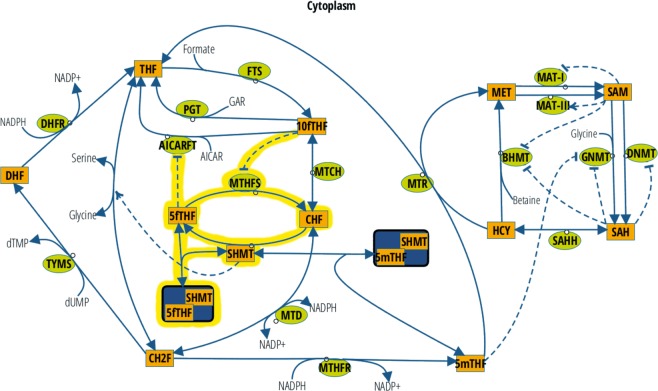
The reaction-based specification of the model according to the notation previously introduced^33^. Orange rectangles identify model variables, non-boxed substrates indicate model constants, green circles identify enzymes (except for SHMT, which is implemented as a model variable). Solid blue arcs identify matter transformation, while dashed blue arcs identify regulatory events (T shaped arrows identify inhibitions and standard arrows identify activations). Additions to the initial model introduced in^17^ are highlighted in yellow.

Our hybrid stochastic model of FOCM showed that decreased 5mTHF binding to SHMT, as a result of an overall decrease in 5mTHF levels resulting from a methylenetetrahydrofolate reductase (*MTHFR*) polymorphism, led to increased flux through the reversible reactions catalyzed by SHMT and MTHFD1^17^. The common *MTHFR* C677T polymorphism is known to lower total cellular MTHFR activity, leading to decreased 5mTHF production and altered one-carbon distribution^18–20^. Decreased 5mTHF levels also increase total reaction propensities, indicating a loss in overall FOCM network stability as a result of this common polymorphism^17^. Here, we extended our hybrid stochastic model of FOCM to include the 5fTHF futile cycle according to Fig. 1 in an effort to better understand: (1) the role of MTHFS and the 5fTHF futile cycle in FOCM, and (2) the relative contributions of 5fTHF and 5mTHF to SHMT activity and overall network stability.

## Materials and Methods

### Mathematical model

The model presented in this paper is an extension of the hybrid stochastic mathematical model of FOCM previously introduced in^17^. The model provides a description of FOCM in the cytoplasm as well as its regulation of key biological processes related to *de novo* dTMP synthesis, *de novo* purine synthesis and remethylation of homocysteine to methionine. With respect to the initial model^17^, the model herein has been extended to include the folate form 5fTHF as well as the enzyme MTHFS and the relevant reactions involving these two molecules, according to the reaction network provided in Fig. 1. The model is composed of 14 variables and 20 (reversible and irreversible) reactions, most of which have been parametrized by means of Michaelis-Menten kinetics with one or two substrates. Whenever possible, physiologically relevant forms of folate polyglutamate cofactors have been considered to derive kinetic coefficients, according to the modeling approach used in the original model^17^. Parameter estimates are detailed in Supplementary Table 2.

MTHFS and 5fTHF have been included in the model using the following five reactions. In agreement with the FOCM modeling literature, ATP and ADP are not explicitly represented in reaction stoichiometry since they are assumed to be constantly present at their physiological level.

1. Formation of 5fTHF catalyzed by MTHFS (Michaelis-Menten kinetics with one substrate)
  $${R}_{MTHFS}:5fTHF\mathop{\to }\limits^{MTHFS}CH{\rm{F}}$$
  $${v}_{MTHFS}=\frac{{k}_{cat}[MTHFS][5fTHF]}{{K}_{m,5fTHF}(1+\frac{[10fTHF]}{{K}_{i,10fTHF}})+[5fTHF]}$$
  The inhibition by 10fTHF has been encoded in the inhibition constant *K*_*i,10fTHF*_.
2. Transformation of 5fTHF to CHF catalyzed by SHMT (Michaelis-Menten kinetics with one substrate)
  $${R}_{SHMT}:CHF\mathop{\to }\limits^{SHMT}5fTHF$$
  $${v}_{SHMT}=\frac{{k}_{cat}[SHMT][CHF]}{{K}_{m,CHF}+[CHF]}$$
3. Tight binding of 5fTHF and SHMT (mass-action kinetics)
  $${R}_{binding}:5fTHF+SHMT\to 5fTHF:SHMT$$
  $${v}_{binding}={k}_{binding}[5fTHF][SHMT]$$
4. Unbinding of 5fTHF: SHMT (mass-action kinetics)
  $${R}_{unbinding}:5fTHF:SHMT\to 5fTHF+SHMT$$
  $${v}_{unbinding}={k}_{unbinding}[5fTHF:SHMT]$$
5. AICARFT mediated 10fTHF transformation to THF (Michaelis-Menten kinetics with two substrates)

$${R}_{AICARFT}:10fTHF+AICAR\mathop{\to }\limits^{AICARFT}THF$$

$${v}_{AICARFT}=\frac{{V}_{max}[10fTHF][AICAR]}{({K}_{m,10fTHF}+[10fTHF])({K}_{m,AICAR}+[AICAR])}\frac{1}{1+\,\frac{[5fTHF]}{{K}_{i,5fTHF}}}$$

This reaction was already included in the initial model^17^, but its rate formula has been extended to account for the inhibition by 5fTHF.

Regarding model initial values, the starting availability of 5fTHF has been set to 5% of total cytosolic folate, according to^16^. Furthermore, the initial value of SHMT has been refined with respect to the value provided in the original hybrid stochastic model^17^ to account for the fact that only two of the four enzyme sites are presumed to be active^21^.

Because the additional components included in the model affect the behavior of the network, some of the reaction kinetics have been refined with respect to those previously provided^17^ to take into account the increased amount of information included in the extended network. All the refined parameter estimates are listed in Supplementary Table 2.

### Simulation approach and computational environment

For the simulation of the model we employed a hybrid-stochastic approach, for which the initial model^17^ and the extensions described above have first been translated to a set of ODEs and implemented in MATLAB. The initial part of the dynamics until reaching a model steady state was computed using deterministic simulation by means of the numerical ODE solver *ode15s*. By exploiting the hybrid stochastic framework, we then coupled the ODE-based model description with a stochastic reaction-based one following the same approach developed for the first version of the mathematical model introduced in^17^. To assess the level of stochasticity of model steady states, we relied on the concept of total propensity, which is used to determine the next reaction event in stochastic simulation^22^. In more detail, the stochastic simulation algorithm computes a propensity function *a*_*j*_(*x*) for each model reaction *R*_*j*_, where x is the current state of the system. The total propensity is then calculated as ${a}_{0}(x)={\sum }_{j}{a}_{j}(x)$ and used to assess when the next reaction event will occur as ${a}_{0}(x)\,\,$is proportional to the number of reaction events occurring per unit of time.

## *In-Silico* Experiments

### The role of MTHFS

The influence of the addition of MTHFS on the FOCM network was studied by comparing steady states and reaction velocities of the standard scenario based on the parameters presented in Supplementary Table 2 with the following four scenarios: a) MTHFS activity scaled down to 50% of the standard case (0.04 µM), b) no availability of MTHFS (0 µM), c) no availability of MTHFS plus turning off of the reaction $\,{R}_{SHMT}\,:\,CHF\mathop{\to }\limits^{SHMT}5fTHF$, d) no inhibition of MTHFS by 10fTHF.

### Effect of glycine and MTHFS activity on 5mTHF binding to SHMT

Because 5fTHF inhibits SHMT activity, the interplay of glycine and MTHFS and their effect on steady state concentrations of 5mTHF (free, bound to SHMT and total) were compared for high and low levels of MTHFS (10% of standard activity and 5-fold increase in standard activity) as a function of glycine (considered levels 1000 µM, 1850 µM, 2000 µM, 5000 µM and 10000 µM). These simulations were repeated for the case in which the binding of 5mTHF and SHMT was blocked, increasing therefore the availability of 5mTHF for other reactions.

### Sensitivity analysis on MTHFS

The effect of MTHFS levels on the binding of 5fTHF and 5mTHF to SHMT was studied by comparing their steady state concentrations at different levels of MTHFS. Enzyme deficiency was simulated by decreasing MTHFS availability stepwise (90% to 10% of the standard activity, corresponding to 0.072 µM and 0.008 µM) whereas the 2-fold and 5-fold increase of the standard activity was considered in overexpression scenarios (corresponding to 0.16 µM and 0.4 µM).

### Quantification of network  stability

In the steady state comparisons herein presented, we exploited the concept of total propensity for comparing the level of stochasticity of the considered steady states as reported in^17^ and in the section “Simulation approach and computational environment”. The idea behind this approach is the following: intuitively, a steady state with lower total propensity can be interpreted as more stable because on average it will have a lower number of reaction events per unit of time that can perturb its equilibrium. We applied this intuition to compare different scenarios and assess the stability of the corresponding steady states. In particular, we investigated the effect of the common C677T MTHFR polymorphism, which reduces the enzymatic activity of MTHFR, and the effect of folate deficiency, to test the impact of these factors on the FOCM network with or without the 5fTHF futile cycle.

Following our previous work in^17^, MTHFR activity was decreased to 30% of standard activity to model the effect of the MTHFR C677T polymorphism. We further considered two levels of folate availability: replete folate status (standard model parametrization) and low folate status (folate availability reduced to 50%). The resulting four scenarios were repeated for the standard parametrization of the model and for the one without the 5fTHF cycle (no availability of MTHFS and 5fTHF, and no activity of the SHMT-catalyzed reaction: CHF → 5fTHF).

In all cases we used deterministic simulation to compute the steady states of the scenarios of interest and we then calculated the reaction propensities and the total propensities according to stochastic simulation.

## Results

### The role of MTHFS

The influence of the 5fTHF futile cycle, including the MTHFS-catalyzed synthesis of CHF from 5fTHF and the reverse reaction catalyzed by SHMT, on the FOCM network was studied by comparing steady states and reaction velocities of this updated model, which is described by the parameters presented in Supplementary Table 2. The outputs of the following four conditions were compared to describe the effects of the 5fTHF futile cycle: a) inclusion of the enzymatic reactions comprising the futile cycle (MTHFS and SHMT), b) MTHFS activity scaled down to 50% of the standard condition, c) no MTHFS activity or d) no activity of either MTHFS or its SHMT-catalyzed counterpart reaction $\,{R}_{SHMT}\,:\,CHF\mathop{\to }\limits^{SHMT}5fTHF$ (elimination of the futile cycle).

In this model that includes the 5fTHF futile cycle, reducing MTHFS activity by 50% leads to increased 5fTHF levels, which come at the expense of 5mTHF levels, though  5mTHF remains the predominant form of intracellular folate under both conditions (Tables 1 and 2, compare conditions “A” and “B”). Levels of other one-carbon substituted folate forms remain unchanged as a result of decreased MTHFS activity (Tables 1 and 2). 5fTHF is a known inhibitor of the folate-dependent enzymes AICARFT and SHMT^15^. We confirm that 5fTHF accumulation resulting from decreased MTHFS activity decreases the flux through the AICARFT-catalyzed reaction (Table 3). Flux through the other folate-dependent *de novo* purine synthesis enzyme, PGT, remains mostly unchanged when MTHFS activity is reduced by 50% (Table 3), which is also not unexpected because 5fTHF is not known to inhibit PGT. Overall, these results are consistent with the empirical observation that “cells with 50% reduced *Mthfs* expression have reduced *de novo* purine synthesis”^10^. Furthermore, the model also supports the empirical observation that *de novo* dTMP synthesis is not affected in cells with reduced *Mthfs* expression, as flux through TYMS and DHFR is not affected by reduced MTHFS activity (Table 3)^10^. The 50% reduction in MTHFS activity moderately decreases fluxes through SHMT-catalyzed reactions (Table 4), and does not affect flux through the enzymes of the homocysteine remethylation cycle (Table 5).

**Table 1:** Steady state concentrations of model variables (in µM) for different scenarios with respect to MTHFS and SHMT activity.

	THF	10fTHF	CHF	CH2F	DHF	5mTHF free	5mTHF bound	5fTHF free	5fTHF bound	SHMT free	HCY	MET	SAM	SAH
Standard MTHFS and regular SHMT activity (Condition “A”)	0.051	7.075	1.407	0.398	0.007	5.217	3.644	0.413	0.577	0.279	3.206	38.116	76.071	33.127
0.5× MTHFS and regular SHMT activity (Condition “B”)	0.047	6.984	1.390	0.394	0.007	4.972	3.249	0.757	0.989	0.261	3.218	38.294	75.278	33.729
0× MTHFS and regular SHMT activity (Condition “C”)	0.000	0.000	0.000	0.000	0.000	0.000	0.000	14.351	4.438	0.062	4.039	40.573	14.010	91.898
0× MTHFS and SHMT: CHF → 5fTHF activity turned off (Condition “D”)	0.058	7.014	1.394	0.391	0.007	4.828	4.156	0.000	0.000	0.344	3.226	38.402	74.790	34.102
Standard MTHFS and regular SHMT activity without MTHFS inhibition by 10fTHF (Condition “E”)	0.056	7.180	1.428	0.403	0.007	5.517	4.194	0.001	0.001	0.304	3.191	37.908	76.973	32.447

**Table 2:** Steady state distribution of folate (in percentage of total folate) for different scenarios with respect to MTHFS and SHMT activity.

	THF	10fTHF	CHF	CH2F	DHF	5mTHF free	5mTHF bound	5mTHF total	5fTHF free	5fTHF bound	5fTHF total
Standard MTHFS and regular SHMT activity (Condition “A”)	0.27	37.65	7.49	2.12	0.04	27.77	19.39	47.16	2.20	3.07	5.27
0.5× MTHFS and regular SHMT activity (Condition “B”)	0.25	37.17	7.40	2.10	0.04	26.46	17.29	43.75	4.03	5.27	9.29
0× MTHFS and regular SHMT activity (Condition “C”)	0.00	0.00	0.00	0.00	0.00	0.00	0.00	0.00	76.38	23.62	100.00
0× MTHFS and SHMT:CHF → 5fTHF activity turned off (Condition “D”)	0.33	39.30	7.81	2.19	0.04	27.05	23.28	50.33	0.00	0.00	0.00
Standard MTHFS and regular SHMT activity without MTHFS inhibition by 10fTHF (Condition “E”)	0.30	38.22	7.60	2.14	0.04	29.36	22.32	51.68	0.01	0.01	0.02

**Table 3:** Steady state fluxes of the reactions catalyzed by the enzymes FTS, MTCH, MTD, MTHFR, GART, AICART, DHFR, TYM S, MTHFR and MTR (in µM/h) for different scenarios with respect to MTHFS and SHMT activity.

	FTS	MTCH: 10fTHF → CHF	MTCH: CHF→ 10fTHF	MTD: CHF→ CH2F	MTD: CH2F→ CHF	MTHFR	PGT	AICARFT	DHFR	TYMS	MTHFS	MTR
Standard MTHFS and regular SHMT activity (Condition “A”)	14281.6	761957.5	758886.0	91831.0	88759.5	23.0	5274.9	5935.2	301.7	301.7	1.9	23.0
0.5 × MTHFS and regular SHMT activity (Condition “B”)	13563.0	754720.3	751813.8	90886.3	87979.8	22.9	5267.2	5389.3	298.7	298.7	1.7	22.9
0 × MTHFS and regular SHMT activity (Condition “C”)	0.0	0.0	0.0	0.0	0.0	0.0	0.0	0.0	0.0	0.0	0.0	0.0
0 × MTHFS and SHMT:CHF → 5fTHF activity turned off (Condition “D”)	15638.3	757127.9	753509.5	91112.6	87494.2	22.9	5269.8	6750.2	296.9	296.9	0.0	22.9
Standard MTHFS and regular SHMT activity without MTHFS inhibition by 10fTHF (Condition “E”)	15326.6	770328.6	767040.0	92922.5	89634.0	23.1	5283.7	6754.4	304.9	304.9	2.1	23.1

**Table 4:** Steady state fluxes of the reactions catalyzed by the enzymes SHMT, the (un-)binding of SHMT and 5mTHF and the (un-) binding of SHMT (in µM/h) for different scenarios with respect to MTHFS and SHMT activity.

	SHMT: CH2F→ THF	SHMT: THF→ CH2F	SHMT: CHF→5fTHF	Binding of 5mTHF & SHMT	Unbinding of 5mTHF:SHMT	Binding of 5fTHF & SHMT	Unbinding of 5fTHF:SHMT
Standard MTHFS and regular SHMT activity (Condition “A”)	3191.9	445.0	1.9	7214.3	7214.3	83.1	83.1
0.5× MTHFS and regular SHMT activity (Condition “B”)	2976.3	391.5	1.7	6433.6	6433.6	142.5	142.5
0× MTHFS and regular SHMT activity (Condition “C”)	0.0	0.0	0.0	0.0	0.0	639.1	639.1
0× MTHFS and SHMT:CHF → 5fTHF activity turned off (Condition “D”)	3910.8	612.2	0.0	8228.4	8228.4	0.0	0.0
Standard MTHFS and regular SHMT activity without MTHFS inhibition by 10fTHF (Condition “E”)	3488.1	527.6	2.1	8305.0	8305.0	0.2	0.2

**Table 5:** Steady state fluxes of the reactions catalyzed by the enzymes BHMT, MAT-I, MAT-III, GNMT, DNMT and SAHH (in µM/h) for different scenarios with respect to MTHFS and SHMT activity.

µM/h	BHMT	MAT-I	MAT-III	GNMT	DNMT	SAHH: SAH→HCY	SAHH: HCY→SAH
Standard MTHFS and regular SHMT activity (Condition “A”)	149.7	111.0	61.7	134.9	123.8	267.5	94.8
0.5× MTHFS and regular SHMT activity (Condition “B”)	150.2	111.5	61.7	140.7	122.7	268.3	95.2
0× MTHFS and regular SHMT activity (Condition “C”)	180.1	129.5	50.6	945.6	23.5	298.9	118.8
0× MTHFS and SHMT:CHF → 5fTHF activity turned off (Condition “D”)	150.5	111.7	61.7	144.4	122.1	268.8	95.4
Standard MTHFS and regular SHMT activity without MTHFS inhibition by 10fTHF (Condition “E”)	149.1	110.5	61.7	128.3	125.0	266.6	94.4

The model also shows that MTHFS activity is necessary to prevent accumulation of cellular folate as 5fTHF. When FOCM was modeled without MTHFS activity, the distribution of folate shifted such that all the folate accumulated as 5fTHF (Tables 1 and 2, comparing conditions “A” and “C”), leaving no folate co-factors available for *de novo* dTMP, purine synthesis (Table 3) or homocysteine remethylation (Tables 4 and 5). Therefore, the model supports the experimental observation that *Mthfs* is an essential gene in mice^10^ and suggests that *MTHFS* expression is necessary to prevent accumulation of 5fTHF and subsequent inhibition of FOCM. If in addition to the deletion of MTHFS activity, the SHMT-catalyzed conversion of CHF to 5fTHF is also turned off thereby effectively modeling the absence of the 5fTHF futile cycle, the lethal pooling of folate as 5fTHF is prevented (Tables 1 and 2, compare “C” to “D”). In addition, because there is no 5fTHF formed in the absence of the futile cycle, the steady state distribution of all remaining one-carbon substituted forms increases (Table 2, compare “A” to “D”). This does not appreciably affect the flux through other FOCM enzymes (MTHFD1, MTHFR, PGT, dihydrofolate reductase (DHFR), thymidylate synthase (TYMS), methionine synthase (MTR)) (Table 3, compare “A” and “D”) or homocysteine remethylation enzymes (Table 5, compare “A” and “D”). As expected, the decrease in 5fTHF levels increases flux through AICARFT (Table 3, compare “A” and “D”) and SHMT (Table 4, compare “A” and “D”) due to loss of inhibition by 5fTHF. The model shows that for the latter, the increase in 5mTHF levels is compensating for the loss of 5fTHF inhibition. Indeed, the amount of SHMT bound by 5mTHF increases from 81% to 92% of total SHMT (Table 1, compare “A” and “D”).

SAM and S-adenosylhomocysteine (SAH) levels are not affected by a 50% reduction in MTHFS activity (Table 1, compare conditions “A” and “B”). However, elimination of MTHFS activity causes SAM levels to decrease by more than 80% and SAH increases by almost 3-fold with respect to the standard condition. The SAM/SAH ratio, which is around 2.3 in the standard case, becomes 0.15 without MTHFS activity (Table 1, compare “A” and “C”). Even if 5mTHF is not available in scenario “C”, because of pooling all cofactors as 5fTHF, the remethylation cycle is active because the model includes the betaine-homocysteine transferase (BHMT)-catalyzed conversion of homocysteine to methionine. This reaction is folate-independent and is not directly affected by 5mTHF levels.

### The role of 10-formylTHF inhibition of MTHFS

10fTHF is a tight-binding inhibitor of MTHFS^13^ creating a mechanism for feedback inhibition of MTHFS such that 5fTHF levels are mobilized when 10fTHF levels are depleted. To understand the role of 10fTHF inhibition of MTHFS, the 10fTHF inhibition term was removed from the model (Tables 1–5, condition “E”). Without inhibition of MTHFS by 10fTHF, increased MTHFS activity leads to depletion of 5fTHF (Tables 1 and 2). Interestingly, the steady-state distribution of folate forms and enzyme fluxes were nearly identical to those resulting from removal of the whole 5fTHF futile cycle (Tables 1–5, compare condition “D” to “E”). These data suggest that 10fTHF inhibition of MTHFS is necessary to maintain 5fTHF levels, which in turn controls *de novo* purine synthesis by inhibiting AICARFT, as shown in cultured cells^15^.

### Effect of glycine and MTHFS activity on 5mTHF binding to SHMT

The SHMT-catalyzed interconversion of serine and glycine is reversible *in vitro* and *in vivo*, and increasing intracellular glycine concentrations has been shown to drive this reaction toward serine synthesis, which consumes CH2F otherwise available for dTMP or SAM synthesis^23^. CH2F exists at a branch point in FOCM and can be used for either synthesis of 5mTHF (through MTHFR) for SAM synthesis or for *de novo* synthesis of dTMP (through TYMS). Glycine has also been shown *in vivo*^23^ and *in silico*^17^ to decrease 5mTHF levels. The relative contributions of 5fTHF and 5mTHF to regulating SHMT activity in response to changes in glycine concentration have not been determined. The interplay of glycine and MTHFS and their respective effects on 5mTHF levels was studied by comparing model steady-state concentrations of 5mTHF (free, bound to SHMT, and total) for high and low levels of MTHFS (10% of standard activity and 5-fold increase in standard activity) as a function of glycine concentration (considered levels 1000 µM, 1850 µM, 2000 µM, 5000 µM and 10000 µM). These simulations were repeated for the case in which the binding of 5mTHF and SHMT was blocked, increasing therefore the availability of 5mTHF for other reactions.

**Figure 2:**
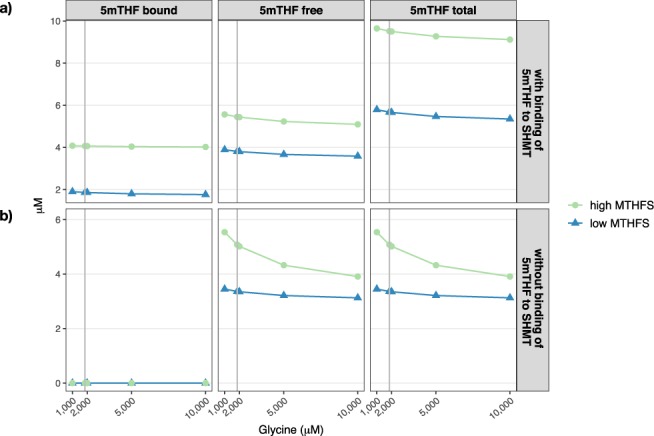
The effect of glycine on the availability of 5mTHF considering low (triangle, blue line) and high (circle, green line) levels of MTHFS activity. (**A**) The model includes binding of 5mTHF to SHMT and (**B**) The model does not include the binding of 5mTHF and SHMT. The grey line represents the concentration of glycine in the “standard” condition.

When running the *in silico* experiment of FOCM, increasing MTHFS activity increased 5mTHF levels by making more folate available for conversion to 5mTHF, but it did not influence the effect that glycine has on 5mTHF levels (Fig. 2A). In this scenario, SHMT is inhibited by both 5fTHF and 5mTHF. Conversely, this is not the case when the binding of 5mTHF to SHMT is removed from the model (Fig. 2B). In this case, 5mTHF levels become responsive to increasing glycine concentration and this decrease in 5mTHF is more pronounced with higher MTHFS activity (Fig. 2B). This is consistent with the observation that a 50% reduction in MTHFS activity and concomitant 2-fold increase in 5fTHF levels (Table 2) do not affect flux through SHMT-catalyzed reactions (Table 4). In other words, intracellular 5fTHF is a meaningful inhibitor of SHMT activity (even when driven by increasing glycine concentrations) but only when 5mTHF levels are low, suggesting that 5mTHF is the predominant intracellular inhibitor of SHMT activity.

### Model sensitivity analysis to unravel the effect of MTHFS activity on FOCM dynamics

Both 5mTHF and 5fTHF bind to SHMT and reduce levels of the free, active form of SHMT. The effect of MTHFS activity on the availability of 5fTHF, and the binding of 5fTHF and 5mTHF to SHMT was studied by comparing model steady-state concentrations of 5fTHF- and 5mTHF-bound SHMT at different levels of MTHFS activity. MTHFS deficiency was simulated by decreasing MTHFS availability stepwise from 90% to 10% of the standard activity whereas the 2-fold and 5-fold increase of the standard activity was considered for overexpression.

**Figure 3:**
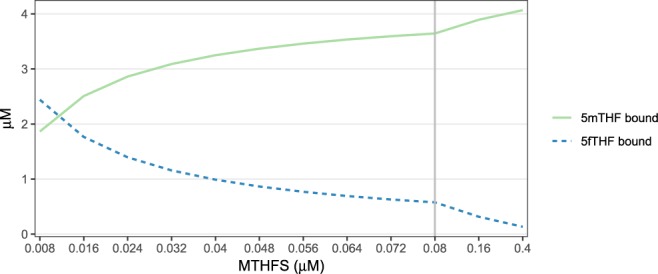
Sensitivity analysis of the relatively effects of changes in MTHFS activity on levels of 5fTHF and 5mTHF bound to SHMT. The blue line represents 5mTHF-bound SHMT and the green line represents 5fTHF-bound SHMT. The grey line represents the concentration of MTHFS in the “standard” condition.

As MTHFS activity increases over a 50-fold range, 5fTHF levels decrease to less than 5% of the initial concentration (Supplementary Table 3). Furthermore, 5mTHF and THF levels increase by about 2-fold with increasing MTHFS activity, and the concentration/distribution of other folate forms is unchanged (Supplementary Tables 3 and 4). Due to the relatively modest changes in levels of one-carbon substituted folates, flux through most FOCM enzymes remains unchanged as a result of elevated MTHFS activity (Supplementary Table 5). Increasing MTHFS activity decreases the amount of 5fTHF that is available to bind and inhibit AICARFT and SHMT (Fig. 3, Supplementary Table 3). As expected, flux through both AICARFT and SHMT increases up to 2-fold as a result of decreased 5fTHF caused by elevated MTHFS activity (Supplementary Tables 5 and 6). These data are consistent with observations in mammalian cells that *MTHFS* expression levels regulate *de novo* purine synthesis^10,13,14^.

Both 5mTHF and 5fTHF are tight-binding inhibitors of SHMT, and interpreting inhibition of SHMT in this scenario where MTHFS activity is increased is complicated by the fact that 5fTHF and 5mTHF levels are affected in opposing directions (5mTHF increases and 5fTHF decreases). However, comparing binding of 5mTHF and 5fTHF as a function of MTHFS activity reveals that there is almost always more 5mTHF than 5fTHF bound to SHMT (Fig. 3). 5fTHF binding to, and inhibition of, SHMT becomes predominant only at very high intracellular 5fTHF concentrations, as would result from an almost 90% reduction in MTHFS activity (Fig. 3). Taken together, these data suggest that 5mTHF is the predominant physiological inhibitor of SHMT and are consistent with the observation that 5fTHF is a meaningful inhibitor of SHMT only when binding of 5mTHF to SHMT is not considered in the model (Fig. 2A,B).

### Quantification of network stability

To understand the effects of the 5fTHF futile cycle on the stability of the FOCM network at steady state, we used the hybrid stochastic framework as described in Materials and Methods. Model steady states were computed both with and without the 5fTHF futile cycle by considering four scenarios regarding two *MTHFR* genotypes (CC, and TT) and two folate levels (replete and low folate), both the *MTHFR* TT genotype and folate deficiency are known to destabilize FOCM.

Interestingly, the addition of the 5fTHF futile cycle reduces the stochastic noise (indicated as lower total reaction propensities) throughout all considered conditions (Table 6, row 1 vs. row 2). Furthermore, the model suggests that the futile cycle mitigates the destabilizing effect that low folate has on the network for the MTHFR CC genotype (Table 6, CC columns). Overall, the SHMT-catalyzed reactions exhibited the greatest stochasticity in response to the absence of 5fTHF futile cycle, the MTHFR C677T polymorphism, and and/or low folate levels (Supplementary Tables 8, 9, 10 and 11). In addition, AICARFT stochasticity also decreased in response to the addition of the futile cycle (Supplementary Table 9).

**Table 6:** Total propensities obtained in eight steady state conditions according to MTHFR polymorphism (CC and TT), folate status (replete, 19 uM; low, 9 uM) and presence of the 5fTHF futile cycle (FOCM network with and without futile cycle).

*a*_0_(*x*)	Replete folate		Low folate	
	CC	TT	CC	TT
With 5fTHF futile cycle	2.95e + 14	5.74e + 14	7.55e + 14	9.56e + 14
Without 5fTHF futile cycle	3.67e + 14	4.21e + 15	1.24e + 15	3.71e + 15

Total propensities can be interpreted as a measure of stability when two steady states are compared since they provide an estimate of how many reactions events occur per unit of time. The steady state with the lower total propensity can be considered more stable than the others.

## Discussion

Folate deficiency and/or genetic and environmental factors that impair FOCM are not only associated with development of pathology but often affect the function of the entire network, which makes it difficult to identify causal pathways associated with these pathologies^24,25^. Computational models advance our understanding of these complex interactions and the effects of perturbations on network function as a whole^24^. In this study, we focused on the role of the 5fTHF futile cycle on the FOCM network function and stability.

The 5fTHF futile cycle was shown to be critical to maintain the stability of the FOCM network, which is a novel finding of this study. Previously, this hybrid stochastic model of FOCM identified both folate deficiency and the common *MTHFR* C677T polymorphism (a known human NTD risk factor through its effects on lowering folate levels^26^) as a source of network instability. The major source of the instability resulted from decreased 5mTHF levels leading to increasing flux through SHMT^17^. In this study, we observed that inclusion of the 5fTHF futile cycle stabilized the FOCM network by introducing 5fTHF inhibition of both SHMT and AICARFT. This stabilization effect of the futile cycle was apparent both when folate deficiency and the MTHFR C677T polymorphism were introduced into the model (Table 6).

The metabolic roles of 5fTHF and MTHFS in mammalian FOCM have not been fully elucidated. 5fTHF does not serve as an enzyme cofactor, rather it acts as an inhibitor of folate-dependent enzymes AICARFT^15^ and SHMT^8^. 5fTHF accumulates in seeds and spores which do not contain MTHFS activity, where 5fTHF is hypothesized to serve as a stable storage form of folate^27^. Conversely, 5fTHF is not known to accumulate in mammalian cells. *Mthfs* is an essential gene in mice^10^, though this is not the case in some prokaryotes nor in *Arabidopsis*^28^. Humans with inborn errors of metabolism in MTHFS exhibit accumulation of 5fTHF in cultured fibroblasts and low cerebrospinal fluid (CSF) folate levels with accompanying neurological sequelae^29^, which are common to many cerebral folate deficiency disorders^30^. Not surprisingly, this updated model of mammalian FOCM indicates that *MTHFS* is essential in mammalian cells to prevent the pooling of cellular folate as 5fTHF (Tables 1 and 2). Such depletion of folate cofactors induced by lack of MTHFS activity drives steady-state flux of all FOCM enzymes to zero  (Tables 3–5). Accordingly, the model also shows that MTHFS is only necessary to prevent 5fTHF pooling when the 5fTHF synthesis activity of SHMT is included in the model (Tables 1 and 2). It is also worth noting that relatively low levels (only 10% of the standard modeled concentration) of MTHFS enzymatic activity are required to prevent this lethal 5fTHF pooling (Supplementary Table 4).

5fTHF and MTHFS levels affect *de novo* purine biosynthesis in cultured cells, although the relative contribution of MTHFS and 5fTHF to the regulation of *de novo* purine synthesis is unknown. The number 2 and number 8 carbons of the purine ring are formed through *de novo* purine synthesis in reactions catalyzed by the enzymes AICARFT and PGT, respectively, which require  the cofactor 10fTHF (Fig. 1). 10fTHF tightly binds to and inhibits MTHFS^13^, and increased MTHFS expression increases 10fTHF levels in cultured cells^15^. 5fTHF also affects *de novo* purine synthesis through its inhibition of AICARFT^15^. Increased *MTHFS* expression increased rates of *de novo* purine synthesis^13^ and caused resistance to antifolates that specifically target *de novo* purine synthesis^14^. The potential mechanisms underlying this observation were suggested to be either: (1) decreased 5fTHF levels and thereby less inhibition of AICARFT, and/or (2) the effect of MTHFS increasing cellular 10fTHF levels. When the 10fTHF inhibition of MTHFS term was removed from the model, folate cofactor distribution (Table 2) and steady-state reaction fluxes of almost all FOCM-dependent enzymes (Tables 3 and 4) were nearly identical to what was observed when the futile cycle was removed from the model. This indicates that 10fTHF inhibition of MTHFS is critical for controlling MTHFS activity and thereby maintaining cellular 5fTHF levels that limit AICARFT activity. It is worth noting that MTHFS has been shown to co-localize with the multi-enzyme *de novo* purine synthesis complex known as the “purinosome”^10^. Purinosomes form when mammalian cells are exposed to purine-deficient culture medium to increase rates of *de novo* purine synthesis^10,31^, leading to the hypothesis that MTHFS delivers or “channels” 10fTHF to the purine synthesis enzymes. This physical interaction adds another layer of regulation among MTHFS activity, 10fTHF, and *de novo* purine synthesis, but the computational model does not yet account for these interactions.

The relative contribution of intracellular 5fTHF and 5mTHF in regulating SHMT, a key enzyme whose deficiency causes folate-responsive NTD risk in mice, has not been investigated to date. Both 5fTHF and 5mTHF tightly bind and inhibit SHMT^15^, and SHMT activity is sensitive to 5mTHF accumulation, as has been demonstrated *in silico*^17^ and in cultured cells^23^. Comparing binding of both 5fTHF and 5mTHF to SHMT as a function of MTHFS activity indicates that 5mTHF is the predominant binder of SHMT (Fig. 3). 5fTHF binding to SHMT becomes predominant only at high levels of intracellular 5fTHF induced by a 90% reduction in MTHFS activity. In other words, 5mTHF (and not 5fTHF) serves as the physiological inhibitor/regulator of SHMT. *De novo* purine biosynthesis is the primary pathway influenced by the 5fTHF futile cycle.

In summary, experimental investigation of the role of the 5fTHF futile cycle has been limited due to the embryonic lethality that occurs in *Mthfs*  knock-out mice. Inclusion of the 5fTHF futile cycle in the *in silico* model has provided new insights into the metabolic functioning of FOCM, by allowing us to investigate conditions that are difficult to reproduce *in vitro* or *in vivo*. The model confirms that loss of MTHFS activity results in accumulation of folate as 5fTHF as occurs in seeds and spores, and replicates observations that the futile cycle impairs *de novo* purine biosynthesis. Importantly, the model provides new mechanistic evidence that the role of MTHFS in accelerating rates of *de novo* purine biosynthesis can be accounted for by its role in lowering 5fTHF levels and alleviating AICARFT inhibition. The model also indicates that the 5fTHF futile cycle plays an important role in limiting the increased stochastic behavior of SHMT introduced by the MTHFR C677T polymorphism and folate deficiency. Importantly, the updated model has identified a potential role of the 5fTHF futile cycle in the etiology of folic acid-responsive NTDs, as the *Shmt1* knock-out mouse is the only folic acid-responsive mouse model of NTDs resulting from disruption of a folate-dependent enzyme^25,32^.

## Supplementary information

Supplementary Information
