# Supplementary material for: The 5-formyltetrahydrofolate futile cycle reduces pathway stochasticity in an extended hybrid-stochastic model of folate-mediated one-carbon metabolism
Source: Sci Rep. 2019 Mar 13;9:4322. doi: 10.1038/s41598-019-40230-4 (PMC6416297; doi:10.1038/s41598-019-40230-4)
Supplement: Supplementary file 1 — Supplementary Information [file 41598_2019_40230_MOESM1_ESM.pdf]

Supplementary Material for

The 5-formyltetrahydrofolate futile cycle reduces pathway stochasticity in an extended hybrid-stochastic model of folate-mediated one-carbon metabolism

Karla Misselbeck[1,2+], Luca Marchetti[1+], Corrado Priami[1,3], Patrick J. Stover[4*], and Martha S. Field[5*]

[1]The Microsoft Research - University of Trento Centre for Computational and Systems Biology (COSBI), Piazza Manifattura, 1, 38068 Rovereto, Italy

[2]Department of Mathematics, University of Trento, 38123 Povo, Italy

[3]Department of Computer Science, University of Pisa, 56127 Pisa, Italy

[4]College of Agriculture and Life Sciences, Texas A&M University, College Station, TX 77843-2142

[5]Division of Nutritional Sciences, Cornell University, Ithaca, NY 14853

[+]these authors contributed equally to this work
[*]these authors contributed equally to this work

Supplementary Table 1: List of Abbreviations and acronyms.

5fTHF	5-formyl tetrahydrofolate
5mTHF	5-methyl tetrahydrofolate
10fTHF	10-formyltetrahydrofolate
AICAR	5-Aminoimidazole-4-carboxamide ribonucleotide
AICARFT	Phosphoribosylaminoimidazolecarboxamide formyltransferase
BHMT	Betaine-homocysteine methyltransferase
CHF	5,10-methenyltetrahydrofolate
CH2F	5,10-methylenetetrahydrofolate
DHF	Dihydrofolate
DHFR	Dihydrofolate reductase
DNMT	DNA methyltransferase
dUMP	Deoxyuridine monophosphate
dTMP	Deoxythymidine monophosphate
FOCM	Folate-mediated one-carbon metabolism
FTD	10-formyltetrahydrofolate dehydrogenase
FTS	Formate-tetrahydrofolate ligase
GAR	Glycinamide ribonucleotide
GNMT	Glycine N-methyltransferase
HCY	Homocysteine
MAT-I	Methionine adenosyltransferase 1
MAT-III	Methionine adenosyltransferase 3
MET	Methionine
MTCH	Methenyltetrahydrofolate cyclohydrolase
MTD	Methylenetetrahydrofolate dehydrogenase
MTHFD	Methylenetetrahydrofolate dehydrogenase
MTHFR	Methylenetetrahydrofolate reductase
MTHFS	Methenyltetrahydrofolate synthetase
MTR	Methionine synthase
NADP+	Nicotinamide adenine dinucleotide phosphate
NADPH	Reduced form of Nicotinamide adenine dinucleotide phosphate
PGT	Phosphoribosylglycinamide formyltransferase
THF	Tetrahydrofolate
TYMS	Thymidylate synthase
SAH	S-adenosyl-homocysteine
SAHH	S-adenosylhomocysteine hydrolase
SAM	S-adenosyl-methionine
SHMT	Serine Hydroxymethyltransferase

Supplementary Table 2: Model parameter estimates (concentrations are expressed in μM, time is expressed in hours). For each value, the reference cell line and the length of the glutamate chain of the associated folate are indicated when available. Parameter estimates not included in the table have been set to the values provided in [1].

**Parameter**	**Metabolite**	**Value**	**Length of glutamate chain**	**Cell line**	**Reference**
R_{MTHFS} : $5fTHF \rightarrow CHF$					
[MTHFS]		0.08			This manuscript, estimated to preserve 5fTHF being 5% of total folate in the steady state [2]
k_{cat}		5400			[3]
K_m	5fTHF	0.2	3		[3], the reference value was halved, because the measurements were made with racemic folates, only half of which are physiological substrates.
K_i	10fTHF	0.015	3		[3], the reference value was halved, because the measurements were made with racemic folates, only half of which are physiological substrates.
R_{SHMT} : $CHF \rightarrow 5fTHF$					
K_{cat}		198			[4]
K_m	CHF	40	4		[4]
(un-)binding of 5fTHF and SHMT					
K_D		0.2		Rabbit liver	[5]
$k_{unbinding}$		144	3	Rabbit liver	[5]
$k_{binding}$		720	3	Rabbit liver	calculated as $k_{binding} = k_{unbinding}/K_D$
$R_{AICARFT}$: $10fTHF \rightarrow THF$					
V_{max}		63350		MCF-7	[6]
K_m	10fTHF	0.3	4-6	Human leukemia	[7]
K_m	AICAR	16.8		Human purH	[8]
K_i	5fTHF	3	5	MCF-7	[9]
(un-)binding of 5mTHF and SHMT					
K_D		0.4	3	Rabbit liver	[5]
$k_{unbinding}$		1980	3	Rabbit liver	[5]
$k_{binding}$		4950	3	Rabbit liver	[5], calculated as $k_{binding} = k_{unbinding}/K_D$

R_{MTR} : $5mTHF + HCY \rightarrow THF + MET$					
V_{max}		26			This manuscript, estimated in the range 0.024 [10] – 500 [11] μM/h
K_m	5mTHF	0.5	6	Pig liver	[10]
K_m	HCY	0.1			[12]

Supplementary Table 3. Steady state concentrations of model variables (in μM) for different levels of MTHFS (ranging from 0.008 μM to 0.4 μM). The results for the standard MTHFS concentration (0.08 μM) are highlighted in light grey.

MTHFS	THF	10fTHF	CHF	CH2F	DHF	5mTHF free	5mTHF bound	5fTHF free	5fTHF bound	SHMT free	HCY	MET	SAM	SAH
**0.008**	0.036	6.477	1.290	0.369	0.007	3.812	1.864	2.495	2.440	0.196	3.287	39.263	70.648	37.322
**0.016**	0.041	6.762	1.346	0.383	0.007	4.421	2.508	1.556	1.765	0.227	3.249	38.727	73.282	35.261
**0.024**	0.043	6.878	1.369	0.389	0.007	4.700	2.862	1.146	1.395	0.244	3.233	38.502	74.335	34.450
**0.032**	0.045	6.943	1.381	0.392	0.007	4.864	3.089	0.910	1.157	0.254	3.224	38.376	74.912	34.009
**0.040**	0.047	6.984	1.390	0.394	0.007	4.972	3.249	0.757	0.989	0.261	3.218	38.294	75.278	33.729
**0.048**	0.048	7.013	1.395	0.395	0.007	5.049	3.368	0.648	0.865	0.267	3.214	38.237	75.533	33.535
**0.056**	0.049	7.034	1.399	0.396	0.007	5.107	3.460	0.567	0.769	0.271	3.211	38.195	75.721	33.393
**0.064**	0.050	7.051	1.403	0.397	0.007	5.152	3.534	0.504	0.692	0.274	3.209	38.163	75.865	33.284
**0.072**	0.050	7.064	1.405	0.398	0.007	5.188	3.594	0.454	0.629	0.277	3.207	38.137	75.979	33.197
**0.080**	0.051	7.075	1.407	0.398	0.007	5.217	3.644	0.413	0.577	0.279	3.206	38.116	76.071	33.127
**0.160**	0.053	7.125	1.417	0.400	0.007	5.359	3.893	0.217	0.316	0.291	3.199	38.016	76.506	32.799
**0.400**	0.055	7.158	1.423	0.402	0.007	5.452	4.067	0.090	0.134	0.298	3.194	37.952	76.783	32.590

Supplementary Table 4. Steady state distribution of folate (in percentage of total folate) for different levels of MTHFS (ranging from 0.008 μM to 0.4 μM). The results for the standard MTHFS concentration (0.08 μM) are highlighted in light grey.

MTHFS	THF	10fTHF	CHF	CH2F	DHF	5mTHF free	5mTHF bound	5mTHF total	5fTHF free	5fTHF bound	5fTHF total
**0.008**	0.19	34.47	6.87	1.96	0.04	20.29	9.92	30.21	13.28	12.99	26.26
**0.016**	0.22	35.99	7.16	2.04	0.04	23.53	13.35	36.87	8.28	9.40	17.68
**0.024**	0.23	36.61	7.29	2.07	0.04	25.02	15.23	40.25	6.10	7.42	13.52
**0.032**	0.24	36.95	7.35	2.09	0.04	25.89	16.44	42.33	4.85	6.16	11.00
**0.040**	0.25	37.17	7.40	2.10	0.04	26.46	17.29	43.75	4.03	5.27	9.29
**0.048**	0.26	37.32	7.43	2.10	0.04	26.87	17.93	44.80	3.45	4.60	8.05
**0.056**	0.26	37.44	7.45	2.11	0.04	27.18	18.42	45.59	3.02	4.09	7.11
**0.064**	0.26	37.53	7.46	2.11	0.04	27.42	18.81	46.23	2.68	3.68	6.37
**0.072**	0.27	37.60	7.48	2.12	0.04	27.61	19.13	46.74	2.42	3.35	5.77
**0.080**	0.27	37.65	7.49	2.12	0.04	27.77	19.39	47.16	2.20	3.07	5.27
**0.160**	0.28	37.92	7.54	2.13	0.04	28.52	20.72	49.24	1.16	1.68	2.84
**0.400**	0.29	38.10	7.58	2.14	0.04	29.02	21.65	50.66	0.48	0.71	1.19

Supplementary Table 5. Steady state fluxes of the reactions catalyzed by the enzymes FTS, MTCH, MTD, MTHFR, GART, AICART, DHFR, TYM S, MTHFR and MTR (in μM/h) for different levels of MTHFS (ranging from 0.008 μM to 0.4 μM). For bidirectional reactions the direction is indicated behind the enzyme name (Enzyme: Substrate → product). The results for the standard MTHFS concentration (0.08 μM) are highlighted in light grey.

MTHFS	FTS	MTCH: 10fTHF → CHF	MTCH: CHF→ 10fTHF	MTD: CHF→ CH2F	MTD: CH2F→ CHF	MTHFR	PGT	AICARFT	DHFR	TYMS	MTHFS	MTR
**0.008**	11141.5	713316.8	711068.9	85479.3	83231.4	22.3	5220.5	3673.1	281.2	281.2	1.2	22.3
**0.016**	12257.1	736798.2	734226.5	88544.9	85973.3	22.7	5247.5	4437.9	291.3	291.3	1.5	22.7
**0.024**	12874.6	746211.2	743475.4	89774.8	87039.0	22.8	5258.0	4880.9	295.2	295.2	1.6	22.8
**0.032**	13277.1	751402.1	748565.0	90452.9	87615.8	22.9	5263.6	5176.4	297.4	297.4	1.7	22.9
**0.040**	13563.0	754720.3	751813.9	90886.3	87979.8	22.9	5267.2	5389.3	298.7	298.7	1.7	22.9
**0.048**	13777.5	757034.3	754077.1	91188.4	88231.3	22.9	5269.7	5550.7	299.7	299.7	1.8	22.9
**0.056**	13944.8	758743.9	755748.1	91411.6	88415.8	23.0	5271.5	5677.5	300.4	300.4	1.8	23.0
**0.064**	14079.1	760060.3	757033.9	91583.4	88557.1	23.0	5272.9	5779.9	300.9	300.9	1.8	23.0
**0.072**	14189.4	761106.1	758055.0	91719.9	88668.8	23.0	5274.0	5864.3	301.3	301.3	1.9	23.0
**0.080**	14281.6	761957.5	758886.0	91831.0	88759.5	23.0	5274.9	5935.2	301.7	301.7	1.9	23.0
**0.160**	14749.0	765971.0	762799.2	92354.5	89182.6	23.1	5279.1	6298.0	303.2	303.2	2.0	23.1
**0.400**	15080.7	768546.7	765306.9	92690.3	89450.5	23.1	5281.8	6559.0	304.2	304.2	2.0	23.1

Supplementary Table 6. Steady state fluxes of the reactions catalyzed by the enzyme SHMT, the (un-)binding of SHMT and 5mTHF and the (un-) binding of SHMT (in μM/h) for different levels of MTHFS (ranging from 0.008 μM to 0.4 μM). For bidirectional reactions the direction is indicated behind the enzyme name (Enzyme: Substrate → product). The results for the standard MTHFS concentration (0.08 μM) are highlighted in light grey.

MTHFS	SHMT: CH2F→ THF	SHMT: THF→ CH2F	SHMT: CHF→5fTHF	Binding of 5mTHF & SHMT	Unbinding of 5mTHF:SHMT	Binding of 5fTHF & SHMT	Unbinding of 5fTHF:SHMT
**0.008**	2177.2	232.8	1.2	3691.5	3691.5	351.4	351.4
**0.016**	2559.3	301.6	1.5	4965.2	4965.2	254.2	254.2
**0.024**	2760.6	342.9	1.6	5665.9	5665.9	200.9	200.9
**0.032**	2887.8	371.0	1.7	6116.9	6116.9	166.6	166.6
**0.040**	2976.3	391.5	1.7	6433.6	6433.6	142.5	142.5
**0.048**	3041.7	407.2	1.8	6669.1	6669.1	124.6	124.6
**0.056**	3092.0	419.6	1.8	6851.3	6851.3	110.7	110.7
**0.064**	3132.1	429.6	1.8	6996.7	6996.7	99.6	99.6
**0.072**	3164.7	438.0	1.9	7115.4	7115.4	90.6	90.6
**0.080**	3191.9	445.0	1.9	7214.3	7214.3	83.1	83.1
**0.160**	3326.8	481.3	2.0	7709.0	7709.0	45.5	45.5
**0.400**	3420.2	507.7	2.0	8053.3	8053.3	19.3	19.3

Supplementary Table 7. Steady state fluxes of the reactions catalyzed by the enzyme BHMT, MAT-I, MAT-III, GNMT, DNMT and SAHH (in μM/h) for different levels of MTHFS (ranging from 0.008 μM to 0.4 μM). For bidirectional reactions the direction is indicated behind the enzyme name (Enzyme: Substrate → product). The results for the standard MTHFS concentration (0.08 μM) are highlighted in light grey.

MTHFS	BHMT	MAT-I	MAT-III	GNMT	DNMT	SAHH: SAH→HCY	SAHH: HCY→SAH
**0.008**	153.1	113.9	61.5	177.0	116.3	272.5	97.1
**0.016**	151.5	112.6	61.6	155.9	120.0	270.2	96.0
**0.024**	150.8	112.0	61.6	147.8	121.4	269.2	95.6
**0.032**	150.5	111.7	61.7	143.5	122.2	268.7	95.3
**0.040**	150.2	111.5	61.7	140.7	122.7	268.3	95.2
**0.048**	150.1	111.3	61.7	138.8	123.1	268.0	95.0
**0.056**	149.9	111.2	61.7	137.4	123.3	267.9	95.0
**0.064**	149.8	111.2	61.7	136.4	123.5	267.7	94.9
**0.072**	149.8	111.1	61.7	135.5	123.7	267.6	94.8
**0.080**	149.7	111.0	61.7	134.9	123.8	267.5	94.8
**0.160**	149.4	110.8	61.7	131.7	124.4	267.1	94.6
**0.400**	149.2	110.6	61.7	129.7	124.8	266.8	94.5

Supplementary Table 8. Stochastic propensities for all model reactions. Reactions are indicated by the enzymes catalyzing them. The propensities are calculated in the model steady state by considering the MTHFR polymorphism (CC and TT), folate status (replete, 19 uM; low, 9 uM) and the presence of the 5fTHF futile cycle (FOCM network with and without futile cycle).

	FTS	MTCH: 10fTHF → CHF	MTCH: CHF→ 10fTHF	MTD: CHF→CH2F	MTD: CH2F→CHF	MTHFR	PGT	AICAR FT	DHFR	TYMS
**Replete folate, with 5fTHF futile cycle, CC**	4.06E+09	2.17E+11	2.16E+11	2.61E+10	2.53E+10	6.55E+06	1.50E+09	1.69E+09	8.58E+07	8.58E+07
**Replete folate, with 5fTHF futile cycle, TT**	4.48E+09	2.83E+11	2.81E+11	3.51E+10	3.35E+10	2.22E+06	1.56E+09	1.29E+09	1.18E+08	1.18E+08
**Replete folate, without 5fTHF futile cycle, CC**	4.45E+09	2.15E+11	2.14E+11	2.59E+10	2.49E+10	6.50E+06	1.50E+09	1.92E+09	8.45E+07	8.45E+07
**Replete folate, without 5fTHF futile cycle, TT**	9.73E+09	3.20E+11	3.14E+11	3.99E+10	3.37E+10	2.23E+06	1.58E+09	1.96E+09	1.19E+08	1.19E+08
**Low folate, with 5fTHF futile cycle, CC**	4.71E+09	1.28E+11	1.26E+11	1.46E+10	1.29E+10	4.51E+06	1.36E+09	1.65E+09	4.19E+07	4.19E+07
**Low folate, with 5fTHF futile cycle, TT**	5.08E+09	1.43E+11	1.41E+11	1.65E+10	1.44E+10	1.45E+06	1.39E+09	1.57E+09	4.70E+07	4.70E+07
**Low folate, without 5fTHF futile cycle, CC**	5.69E+09	1.36E+11	1.34E+11	1.56E+10	1.31E+10	4.56E+06	1.38E+09	1.86E+09	4.27E+07	4.27E+07
**Low folate, without 5fTHF futile cycle, TT**	8.46E+09	1.91E+11	1.85E+11	2.21E+10	1.70E+10	1.60E+06	1.47E+09	1.91E+09	5.61E+07	5.61E+07

	SHMT: CH2F→ THF	SHMT: THF→ CH2F	SHMT: CHF→ 5fTHF	Binding of 5mTHF & SHMT	Unbinding of 5mTHF:SHMT	Binding of 5fTHF & SHMT	Unbinding of 5fTHF:SHMT	MTHFS	MTR
**Replete folate, with 5fTHF futile cycle, CC**	2.58E+14	3.60E+13	5.35E+05	2.05E+09	2.05E+09	2.36E+07	2.36E+07	5.35E+05	6.55E+06
**Replete folate, with 5fTHF futile cycle, TT**	5.02E+14	7.11E+13	1.34E+06	1.54E+08	1.54E+08	1.53E+08	1.53E+08	1.34E+06	2.22E+06
**Replete folate, without 5fTHF futile cycle, CC**	3.17E+14	4.96E+13	0	2.34E+09	2.34E+09	0	0	0	6.50E+06

**Replete folate, without 5fTHF futile cycle, TT**	2.97E+15	1.24E+15	0	9.09E+08	9.09E+08	0	0	0	2.23E+06
**Low folate, with 5fTHF futile cycle, CC**	6.13E+14	1.42E+14	9.09E+05	1.08E+09	1.08E+09	6.82E+07	6.82E+07	9.09E+05	4.51E+06
**Low folate, with 5fTHF futile cycle, TT**	7.71E+14	1.84E+14	1.23E+06	1.92E+08	1.92E+08	1.26E+08	1.26E+08	1.23E+06	1.45E+06
**Low folate, without 5fTHF futile cycle, CC**	9.63E+14	2.80E+14	0	1.73E+09	1.73E+09	0	0	0	4.56E+06
**Low folate, without 5fTHF futile cycle, TT**	2.57E+15	1.14E+15	0	6.65E+08	6.65E+08	0	0	0	1.60E+06

	**SAHH: SAH→ HCY**	**SAHH: HCY→ SAH**	**BHMT**	**MAT-I**	**MAT-III**	**GNMT**	**DNMT**
**Replete folate, with 5fTHF futile cycle, CC**	7.61E+07	2.70E+07	4.26E+07	3.16E+07	6.49E+07	3.95E+07	3.52E+07
**Replete folate, with 5fTHF futile cycle, TT**	8.44E+07	3.24E+07	4.97E+07	3.68E+07	5.22E+07	2.27E+08	1.10E+07
**Replete folate, without 5fTHF futile cycle, CC**	7.65E+07	2.71E+07	4.28E+07	3.18E+07	6.45E+07	4.23E+07	3.47E+07
**Replete folate, without 5fTHF futile cycle, TT**	8.44E+07	3.24E+07	4.97E+07	3.68E+07	5.22E+07	2.27E+08	1.10E+07
**Low folate, with 5fTHF futile cycle, CC**	8.26E+07	3.06E+07	4.76E+07	3.58E+07	5.55E+07	1.47E+08	1.96E+07
**Low folate, with 5fTHF futile cycle, TT**	8.47E+07	3.29E+07	5.03E+07	3.69E+07	5.17E+07	2.46E+08	9.19E+06
**Low folate, without 5fTHF futile cycle, CC**	8.26E+07	3.05E+07	4.75E+07	3.58E+07	5.56E+07	1.45E+08	1.99E+07
**Low folate, without 5fTHF futile cycle, TT**	8.46E+07	3.28E+07	5.02E+07	3.69E+07	5.18E+07	2.42E+08	9.51E+06

Supplementary Table 9. Comparison between the propensities provided in Supplementary Table 8 with respect to the presence/absence of the 5fTHF futile cycle. The difference propensity_without_futile_cycle - propensity_with_futile_cycle is expressed in % of the scenario without futile cycle.

%	FTS	MTCH: 10fTHF → CHF	MTCH: CHF→ 10fTHF	MTD: CHF→ CH2F	MTD: CH2F→ CHF	MTHFR	PGT	AICARFT	DHFR	TYMS
**Replete folate, CC**	8.68	-0.64	-0.71	-0.79	-1.45	-0.68	-0.10	12.07	-1.59	-1.59
**Replete folate, TT**	53.94	11.72	10.50	11.99	0.67	0.27	1.42	34.07	0.76	0.76
**Low folate, CC**	17.16	6.33	5.89	6.24	1.74	1.11	1.48	11.31	1.82	1.82
**Low folate, TT**	40.02	24.68	23.75	25.33	15.42	9.44	5.29	17.56	16.24	16.24
	**SHMT: CH2F→ THF**	**SHMT: THF→ CH2F**	**SHMT: CHF→ 5fTHF**	**Binding of 5mTHF & SHMT**	**Unbinding of 5mTHF:SHMT**	**Binding of 5fTHF & SHMT**	**Unbinding of 5fTHF:SHMT**	**MTHFS**	**MTR**	
**Replete folate, CC**	18.38	27.32	NaN	12.32	12.32	NaN	NaN	NaN	-0.68	
**Replete folate, TT**	83.08	94.27	NaN	83.11	83.11	NaN	NaN	NaN	0.27	
**Low folate, CC**	36.34	49.41	NaN	37.62	37.62	NaN	NaN	NaN	1.11	
**Low folate, TT**	70.01	83.86	NaN	71.15	71.15	NaN	NaN	NaN	9.44	
%	**SAHH: SAH→ HCY**	**SAHH: HCY→ SAH**	**BHMT**	**MAT-I**	**MAT-III**	**GNMT**	**DNMT**			
**Replete folate, CC**	0.47	0.61	0.56	0.63	-0.57	6.51	-1.43			
**Replete folate, TT**	0.00	-0.01	-0.01	0.00	0.01	-0.07	0.14			
**Low folate, CC**	-0.08	-0.17	-0.14	-0.13	0.22	-1.54	1.34			
**Low folate, TT**	-0.06	-0.29	-0.21	-0.02	0.19	-1.38	3.40			

Supplementary Table 10. Comparison between the propensities provided in Supplementary Table 8 regarding the MTHFR polymorphism. The difference propensity_CC - propensity_TT is expressed in % of CC.

%	FTS	MTCH: 10fTHF → CHF	MTCH: CHF→ 10fTHF	MTD: CHF→ CH2F	MTD: CH2F→ CHF	MTHFR	PGT	AICARFT	DHFR	TYMS
**Replete folate, with futile cycle**	10.28	30.45	30.22	34.49	32.67	-66.04	3.70	-23.63	37.03	37.03
**Replete folate, without futile cycle**	118.66	48.72	46.55	54.01	35.50	-65.71	5.30	1.85	40.28	40.28
**Low folate, with futile cycle**	-7.75	-12.27	-12.12	-12.92	-11.47	67.89	-2.63	4.75	-12.08	-12.08
**Low folate, without futile cycle**	-48.83	-39.62	-38.38	-41.79	-29.51	64.94	-6.75	-2.46	-31.38	-31.38

%	SHMT: CH2F→ THF	SHMT: THF→ CH2F	SHMT: CHF→ 5fTHF	Binding of 5mTHF & SHMT	Unbinding of 5mTHF:SHMT	Binding of 5fTHF & SHMT	Unbinding of 5fTHF:SHMT	MTHFS	MTR
**Replete folate, with futile cycle**	94.39	97.31	151.39	-92.52	-92.52	547.76	547.76	151.39	-66.04
**Replete folate, without futile cycle**	837.57	2402.38	NaN	-61.16	-61.16	NaN	NaN	NaN	-65.71
**Low folate, with futile cycle**	-25.78	-30.06	-35.39	82.24	82.24	-84.55	-84.55	-35.39	67.89
**Low folate, without futile cycle**	-167.01	-307.64	NaN	61.60	61.60	NaN	NaN	NaN	64.94

%	SAHH: SAH→ HCY	SAHH: HCY→ SAH	BHMT	MAT-I	MAT-III	GNMT	DNMT
**Replete folate, with futile cycle**	10.82	20.14	16.73	16.57	-19.48	474.50	-68.74
**Replete folate, without futile cycle**	10.30	19.40	16.07	15.83	-19.02	436.72	-68.25
**Low folate, with futile cycle**	-2.45	-7.68	-5.76	-3.01	6.96	-66.96	53.23
**Low folate, without futile cycle**	-2.47	-7.54	-5.68	-3.13	6.99	-67.23	52.23

Supplementary Table 11. Comparison between the propensities provided in Supplementary Table 8 with respect to folate status. The difference propensity_replete_folate – propensity_low_folate is expressed in % of replete folate.

%	FTS	MTCH: 10fTHF → CHF	MTCH: CHF→ 10fTHF	MTD: CHF→ CH2F	MTD: CH2F→ CHF	MTHFR	PGT	AICARFT	DHFR	TYMS
**with futile cycle, CC**	-15.93	41.05	41.60	44.05	48.86	31.16	9.62	2.27	51.18	51.18
**with futile cycle, TT**	-13.26	49.27	49.72	53.02	57.03	34.92	10.55	-21.90	60.07	60.07
**without futile cycle, CC**	-27.80	36.67	37.50	39.85	47.20	29.91	8.17	3.11	49.49	49.49
**without futile cycle, TT**	13.02	40.54	40.99	44.63	49.54	28.33	6.90	2.52	52.69	52.69

%	SHMT: CH2F → THF	SHMT: THF→ CH2F	SHMT: CHF→ 5fTHF	Binding of 5mTHF & SHMT	Unbinding of 5mTHF:SHMT	Binding of 5fTHF & SHMT	Unbinding of 5fTHF:SHMT	MTHFS	MTR
**with futile cycle, CC**	-137.29	-293.08	-70.00	47.37	47.37	-188.62	-188.62	-70.00	31.16
**with futile cycle, TT**	-53.53	-159.11	8.44	-24.89	-24.89	17.77	17.77	8.44	34.92
**without futile cycle, CC**	-204.21	-464.71	NaN	26.03	26.03	NaN	NaN	NaN	29.91
**without futile cycle, TT**	13.36	8.01	NaN	26.86	26.86	NaN	NaN	NaN	28.33

%	SAHH: SAH→ HCY	SAHH: HCY→ SAH	BHMT	MAT-I	MAT-III	GNMT	DNMT
**with futile cycle, CC**	-8.55	-13.33	-11.63	-13.37	14.40	-272.39	44.24
**with futile cycle, TT**	-0.35	-1.58	-1.13	-0.18	1.09	-8.22	16.58
**without futile cycle, CC**	-7.96	-12.45	-10.86	-12.51	13.72	-242.85	42.67
**without futile cycle, TT**	-0.30	-1.29	-0.93	-0.17	0.91	-6.82	13.76

References

[1] K. Misselbeck, L. Marchetti, M. S. Field, M. Scotti, C. Priami, and P. J. Stover, "A hybrid stochastic model of folate-mediated one-carbon metabolism: Effect of the common C677T MTHFR variant on de novo thymidylate biosynthesis," *Sci. Rep.*, vol. 7, 2017.

[2] S. Girgis, J. R. Suh, J. Jolivet, and P. J. Stover, "5-Formyltetrahydrofolate Regulates Homocystein Remethylation in Human Neuroblastoma.," *J. Biol. Chem.*, vol. 272, no. 8, pp. 4729–4734, 1997.

[3] M. S. Field, D. M. E. Szebenyi, and P. J. Stover, "Regulation of de novo purine biosynthesis by methenyltetrahydrofolate synthetase in neuroblastoma," *J. Biol. Chem.*, vol. 281, no. 7, pp. 4215–4221, 2006.

[4] P. J. Stover and V. Schirch, "Enzymatic mechanism for the hydrolysis of 5,10-methenyltetrahydropteroylglutamate to 5-formyltetrahydropteroylglutamate by serine hydroxymethyltransferase," *Biochemistry*, vol. 31, no. 7, pp. 2155–2164, 1992.

[5] P. J. Stover and V. Schirch, "5-Formyltetrahydrofolate Polyglutamates Are Slow Tight Binding Inhibitors of Serine Hydroxymethyltransferase," *J. Biol. Chem.*, vol. 266, no. 3, pp. 1543–1550, 1991.

[6] P. F. Morrison and C. J. Allegra, "Folate cycle kinetics in human breast cancer cells," *J Biol Chem*, vol. 264, no. 18, pp. 10552–10566, 1989.

[7] J. Thorndike, Y. Gaumont, R. L. Kisliuk, F. M. Sirotnak, B. R. Murthy, M. G. Nair, and J. R. Piper, "Inhibition of glycinamide ribonucleotide formyltransferase and other folate enzymes by homofolate polyglutamates in human lymphoma and murine leukemia cell extracts," *Cancer Res.*, vol. 49, pp. 158–163, 1989.

[8] E. Rayl, B. Moroson, and P. Beardsley, "The Human purH Gene Product , 5-Aminoimidazole-4-carboxamide Ribonucleotide Formyltransferase / IMP Cyclohydrolase," *J. Biol. Chem.*, vol. 271, no. 4, pp. 2225–2233, 1996.

[9] R. Bertrand and J. Jolivet, "Methenyltetrahydrofolate synthetase prevents the inhibition of phosphoribosyl 5-aminoimidazole 4-carboxamide ribonucleotide formyltransferase by 5-formyltetrahydrofolate polyglutamates," *J. Biol. Chem.*, vol. 264, no. 15, pp. 8843–8846, 1989.

[10] R. G. Matthews, C. Ghose, J. M. Green, K. D. Matthews, and R. Bruce Dunlap, "Folylpolyglutamates as substrates and inhibitors of folate-dependent enzymes," *Adv. Enzyme Regul.*, vol. 26, pp. 157–171, 1987.

[11] M. C. Reed, R. L. Thomas, J. Pavisic, S. J. James, C. M. Ulrich, and H. F. Nijhout, "A mathematical model of glutathione metabolism.," *Theor. Biol. Med. Model.*, vol. 5, no. 9, p. 8, 2008.

[12] M. C. Reed, H. F. Nijhout, M. L. Neuhouser, J. F. Gregory III, B. Shane, S. J. James, A. Boynton, and C. M. Ulrich, "A Mathematical Model Gives Insights into Nutritional and Genetic Aspects of Folate-Mediated One-Carbon Metabolism," *J. Nutr.*, vol. 136, no. 10, pp. 2653–2661, 2006.
